# Clinical Implications of a Novel, Iron-containing Fiducial Marker in Radiotherapy for Liver Tumors: An Initial Experience

**DOI:** 10.7759/cureus.1902

**Published:** 2017-12-01

**Authors:** Hiroshi Doi, Shogo Harui, Hiroki Nakajima, Akira Ando, Keiji Kamino, Masayuki Fujiwara, Takayoshi Nakajima, Shinichi Ikura, Tsukasa Aihara, Naoki Yamanaka

**Affiliations:** 1 Department of Radiology, Hyogo College of Medicine; 2 Department of Radiation Oncology, Meiwa Cancer Clinic; 3 Department of Surgery, Meiwa Hospital

**Keywords:** stereotactic body radiation therapy, intensity modulated radiotherapy, image guided radiation therapy, stereotactic ablative radiation therapy, liver metastasis, hepatocellular carcinoma, fiducial marker

## Abstract

A 0.5%-iron-containing fiducial marker, Gold Anchor^TM^ (Naslund Medical AB, Huddinge, Sweden), has been recently developed. Herein, we report our initial experiences with the clinical use of the Gold Anchor^TM ^(GA) in radiotherapy for liver tumors. Data of four consecutive patients with liver tumors, including two liver metastases and two hepatocellular carcinomas, were retrospectively analyzed. The GA was percutaneously placed under local anesthesia, close to the tumor. Gadolinium-ethoxybenzyl-diethylenetriamine pentaacetic acid-enhanced magnetic resonance imaging (MRI) was performed after the placement of the GA. Radiotherapy was designed using the volumetric modulated arc therapy technique. All procedures for placement of the GA were successfully performed with no complications. The GA exhibited various forms in the liver in the four patients. All of the GAs were well-detected on MRI, planned computed tomography (CT), and cone-beam CT. Additionally, the tadpole-like shape of the GA showed better detectability than the uptake of lipiodol emulsion and could be used for three-dimensional correlation during setup in daily image-guided radiotherapy. GA was a useful tool in image registration of radiotherapy with a high applicability. Additionally, the tadpole-like shape can be recommended for liver radiotherapy. Our findings suggest that the GA will indeed be useful in clinical practice.

## Introduction

Modern radiotherapy techniques, including intensity-modulated radiation therapy (IMRT) and stereotactic body radiation therapy (SBRT), have recently been frequently utilized to curatively treat liver tumors. SBRT, also known as stereotactic ablative radiation therapy (SABR), delivers highly conformal radiation in a limited number of high-dose fractions, provides excellent primary tumor control with minimal toxicity, and is a proven curative treatment for small inoperable liver tumors [1-2].

The insertion of a fiducial marker near the tumor before radiotherapy allows respiratory motion to be tracked; thus, enabling accurate dose delivery while the patient breathes freely [3]. Recently, the percutaneous insertion of fiducial markers has been described [4-6].

To ensure accurate radiation beam delivery, stereotactic localization of the target is performed for each treatment with imaging and/or placement of fiducial markers. In the case of SBRT, IMRT, and image-guided radiotherapy (IGRT), such as delivery systems that have an onboard computed tomography (CT), cone-beam CT (CB-CT) is needed to confirm the location of a tumor immediately before and during the delivery of radiation. For either localization system, visible tumors, fiducial markers, or appropriate anatomic landmarks are used with the aim to repeat the treatment.

A 0.5%-iron-containing fiducial marker has been recently reported to have a high visibility in magnetic resonance imaging (MRI) and is possibly superior to markers containing no iron [7-9]. Markers capable of being depicted on MRI can improve contouring of the tumor during treatment planning as liver tumors are often ill-defined on CT. However, to the best of our knowledge, only a few reports have reported the clinical implications of the 0.5%-iron-containing fiducial marker in radiotherapy for liver tumors.

In this study, we report our findings regarding the utility of a 0.5%-iron-containing fiducial marker, Gold Anchor^TM^ (GA; Naslund Medical AB, Huddinge, Sweden) in radiotherapy for liver tumors from our initial experience.

## Technical report

Our institutional review board approved data collection and analysis (Approval No. 28–35). From June 2016 to March 2017, the data of four consecutive patients were retrospectively analyzed in this study (Table 1). All of the patients provided written informed consent prior to treatment.

**Table 1:** Patients’ characteristics

Case	Age (in years)	Sex	Tumor location	Maximum diameter of tumor (mm)	Tumor	Planned radiotherapy
1	81	Male	S4	50	Hepatocellular carcinoma	44 Gy/4 fr
2	88	Female	S4	61	Metastatic liver tumor (ascending colon cancer)	72 Gy/30 fr
3	70	Male	S8	10	Metastatic liver tumor (cecal cancer)	60 Gy/8 fr
4	65	Male	S2	14	Hepatocellular carcinoma	44 Gy/4 fr

The fiducial marker was percutaneously placed, under local anesthesia, near the tumor by a hepatobiliary surgeon. The GA was 0.28 mm in diameter, 20 mm in length, and had a shape that could be bent to fold the marker [8-9].

MRI was performed on a 1.5T Philips Gyroscan Intera scanner (Philips Medical Systems, Cleveland, OH, USA) one week after the implantation of the GA.

Patients were placed in the supine position and scanned using an Aquilion LB CT unit (Toshiba, Ohtawara, Japan) with immobilization devices. The CT images were reconstructed with a slice thickness of 2 mm. The acquired CT dataset was transferred to the Pinnacle^3^ treatment planning system (Philips Radiation Oncology Systems, Fitchburg, WI) or the XiO treatment planning system (Elekta Inc., Stockholm, Sweden) and the volumes of interest were outlined.

Planning contrast-enhanced four-dimensional CT scans and gadolinium-ethoxybenzyl-diethylenetriamine pentaacetic acid (Gd-EOB-DTPA)-enhanced MRI images were used to determine gross tumor volume. To account for respiratory tumor motion, an internal target volume (ITV) was generated by contouring the imaging data of the four-dimensional CT. Planning target volume (PTV) was created by adding 5 mm margins to the ITV in all directions. The prescription radiation doses were designed to deliver the prescription dose to cover 95% of the PTV in SBRT and to deliver the mean dose of the prescription dose for PTV in conventional radiotherapy using IMRT. IMRT was administered using volumetric modulated arc therapy (VMAT). VMAT plans were created using the Pinnacle^3^ treatment planning system (Philips Radiation Oncology Systems, Fitchburg, WI, USA) and performed using a Synergy linear accelerator (Elekta Ltd., Crawley, UK).

**Figure 1:**
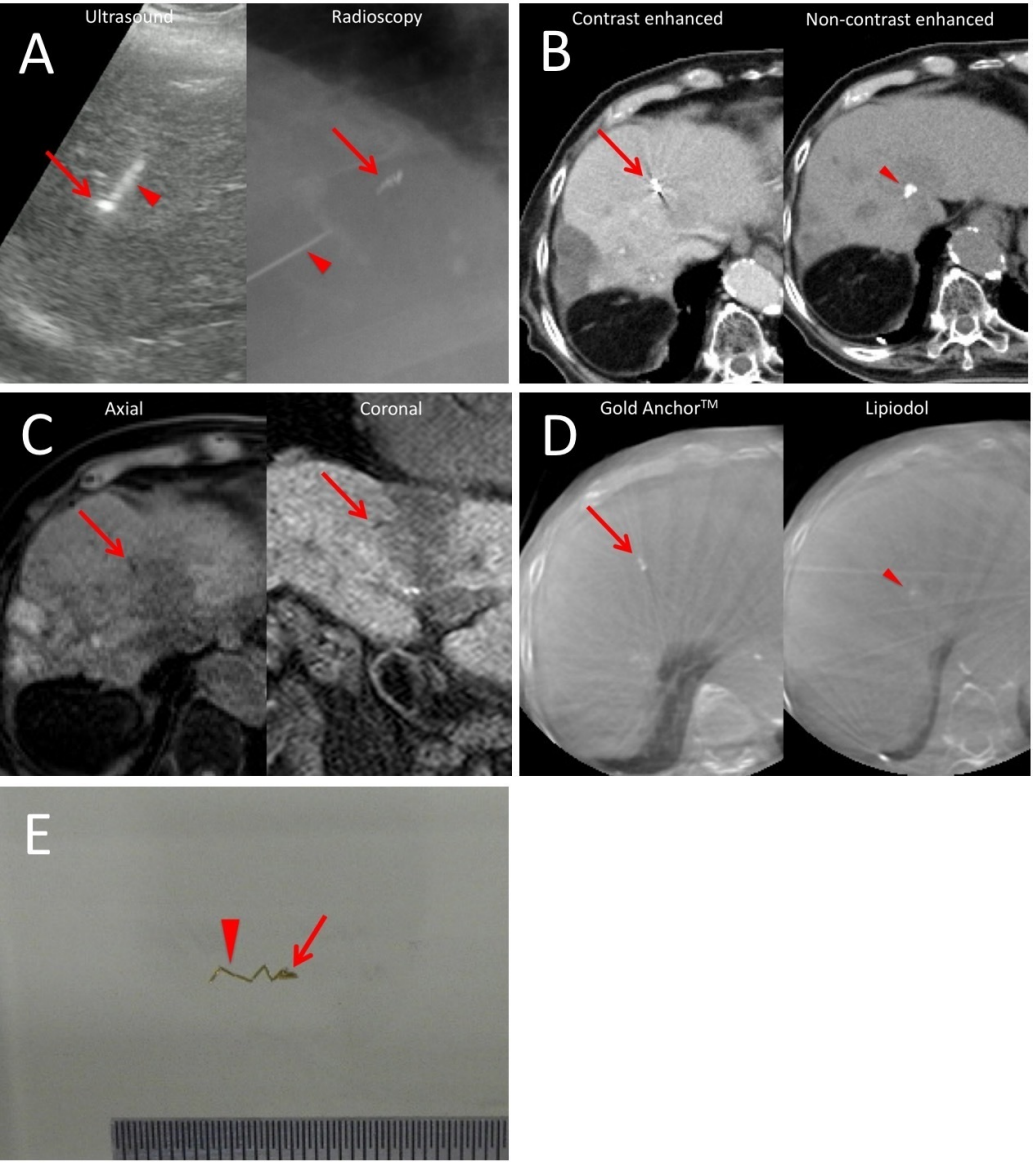
Placement of the GA; the GA in radiographic images. These images show the placement of the Gold Anchor^TM ^(GA) in an 81-year old man with recurrent hepatocellular carcinoma after 11 rounds of radiofrequency ablation and eight rounds of transcatheter arterial chemoembolization. The Gold Anchor^TM^ (GA) was percutaneously injected into the liver close to the tumor under ultrasound and radiographic guidance (A). The arrow and arrowhead indicate the GA and the needle, respectively. The GA showed the tadpole-like shape. The GA was well-detected in both images on computed tomography (CT) (B, left) and gadolinium-ethoxybenzyl-diethylenetriamine pentaacetic acid (Gd-EOB-DTPA)-enhanced magnetic resonance imaging (MRI) (C). The uptake of lipiodol emulsion was also well-detected on CT (B, right, arrowhead). In cone-beam CT (CB-CT) for image-guided radiotherapy (IGRT), the GA (arrow) showed a better detectability than the uptake of lipiodol emulsion (arrowhead) (D). The tadpole-like shape of the GA (E). A folded shape (arrow) followed by a linear shape (arrowhead). GA = Gold Anchor^TM^

All procedures for placement of the GA were successfully performed with no complications. Images of the GA from placement through to treatment (Case 1) are shown as a brief case report in Figure 1.

**Figure 2:**
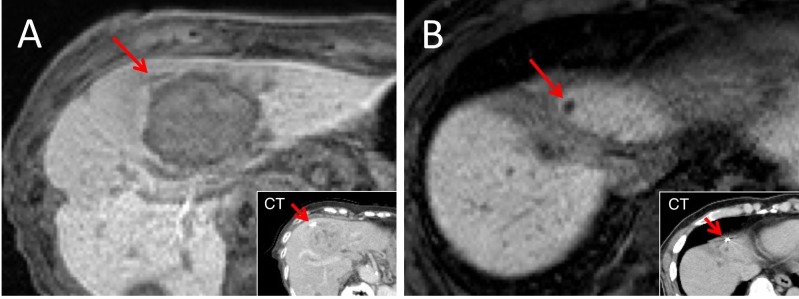
Various forms of the GA in CT and MRI images. These images show the Gold Anchor^TM^ (GA) in contrast-enhanced computed tomography (CT) and gadolinium-ethoxybenzyl-diethylenetriamine pentaacetic acid (Gd-EOB-DTPA)-enhanced magnetic resonance imaging (MRI) images. A. The GA was placed close to the tumor in a linear shape. B. The GA was placed near the postoperative site in a folded shape. GA = Gold Anchor^TM^

The GA showed various forms (Figure 2) and was well detected in CB-CT images in all fractions of the radiotherapy course except in one patient who refused radiotherapy after planning CT (Case 2).

## Discussion

A flexible marker can be a useful tool in modern radiotherapy, which requires high precision during the treatment course. The flexible gold fiducial markers have been used to minimize the uncertainty regarding the positions of liver tumors in radiotherapy, especially for target gating or target chasing [6, 10-13]. In tumor-tracking SBRT, the movements of the skin surface or other markers are monitored during irradiation.

Bertholet, et al. have described how the distance of three implanted markers in liver SBRT has a positive correlation with the root-mean-square error as well as the importance of the rotational correction for improvement of the localization accuracy [14]. Multiple markers provide better visibility and greater correction of setup errors because of the ability for three-dimensional evaluation. However, in comparison with single marker placement, multiple marker placements are more intensive and can be less constant because multiple punctures can lead to human errors in the location of the markers. Since there might be a potential risk of tumor implantation, fewer punctures may be valuable from this point of view [15]. In addition, a small number of fiducial markers can be cost-effective in clinical practice. Moreover, the GA can be used with needles as thin as 25 G. Mueller, et al. showed a clear increase in bleeding complications associated with the use of thicker needles [16]. Therefore, the use of GA can be safer because of the thinness of the needles.

The GA can be placed in a folded shape and made linear later. The GA has this novel character and can provide a folded shape followed by a linear shape (the tadpole-like shape) in the organ (Figure 1). The tadpole-like shape has several features. First, multiple assessable points are provided at once by a single marker. Second, the GA contains 0.5% iron and is highly visible in the liver on MRI. The GA used in the present study contains 0.5% iron, and its visibility on MRI has been reported to be superior to that of markers containing no iron [7-9]. Tanaka, et al. have directly compared the visibility of the GA with a marker containing no iron in the same patient with prostate cancer and reported the superiority of the GA in terms of the detectability on MRI compared with a marker containing no iron in five patients [8-9]. We assessed four patients with liver tumors as an initial report here; further clinical study is needed in order to compare the possible benefits from the features of the GA objectively.

Modern radiotherapy, including SBRT and IMRT, can provide precipitous radiation dose distribution and requires the reproducibility of high-precision techniques throughout the radiotherapy course since we irradiate the target while reducing the dosage to the surrounding normal tissues. Those techniques could improve dose distribution of radiotherapy and contribute to improved clinical outcomes. Visibility of the target in contouring and the radiotherapy course is important since precipitous radiation dose distribution might increase marginal recurrence after treatment. Since the outlines of liver tumors are indistinct, and treatment adjustment and contouring of the organs can prove difficult when using CT alone, MRI is often fused with planning CT images in order to compensate for any shortcomings of CT. We presented the good visibility of the GA in radiographic images, including CT, MRI, and CB-CT, which are routinely used in clinical practices.

We showed that the GA was more visible than the uptake of lipiodol emulsion in this study. The lipiodol emulsion is well-detected on CT images, and we hypothesized that its visibility could surpass that of fiducial markers in liver radiotherapy. However, the use of the GA yielded greater visibility in this case (case 1, Figure 1E). To the best of our knowledge, this is the first report to compare the visibility of the GA and the uptake of lipiodol emulsion.

The recognition precision on MRI increases with marker size, and we presented various forms of the GA in the liver in Figure 2. As a result, both linear and folded shapes of the GA were well-identified on Gd-EOB-DTPA-enhanced MRI. The tadpole-like shape of the GA had the advantage of two different shapes, linear and folded. The linear shape can provide three-dimensional information in radiological images and the folded shape of the GA is well-defined on MRI. The utility of the GA has been reported in radiotherapy for prostate cancer. As the prostate is a relatively small organ, it can be challenging to make linear and tadpole-like shapes in markers placed there. However, the high flexibility of the GA in placement is utilized in radiotherapy for liver tumors. Therefore, we suggest that the tadpole-like shape can be highly recommended for liver radiotherapy.

In addition, the presence of metal, either in the marker or within the organ itself, may influence the dose distribution. Tanaka, et al. reported an optimal MRI sequence based on marker size for prostates. Iron-containing markers have been approved for clinical use in Japan since February 2016. Most fiducial markers have a 0.35- to 0.75-mm-diameter; however, the GA with a 0.28-mm marker was well-recognized on CB-CT and might help reduce the artifacts on CT because of the small size.

To our knowledge, no previous studies have shown various forms of the GA in livers. Nevertheless, in this initial experience, we found that the tadpole-like shape could be the best shape in radiotherapy for liver tumor. Some complications such as marker migration have been reported after percutaneous fiducial marker placement, which might cause delayed or inappropriate treatment [4-5, 12]. We hypothesize that the GA can reduce the complications because of the unique features, including the thin size and the flexibility of the three-dimensional forms that it can take. In addition, the shape may prevent migration. Further clinical studies with larger number of patients and longer follow-up would be required in order to substantiate the evidence of the utility of the GA and to develop the ideal techniques of placement. Although the GA, with its thinness, might reduce metal artifacts and scattering radiation, the various stereoscopic shapes of the GA might lead to variable results. In addition, VMAT, which delivers beams from numerous directions, may avoid strong artifacts from artificial materials in comparison with other radiotherapeutic modalities using fewer beams. Therefore, basic research, including phantom analysis, is under investigation with the aim of physically validating the impact of the GA on the clinical treatment.

## Conclusions

Our findings show that an iron-containing marker, the GA, is useful in image registration, including CB-CT and MRI, and that the tadpole-like shape can be recommended for liver radiotherapy. The present findings suggest that the GA will indeed be useful in clinical practice.
